# Possible Abscopal Effect Observed in Frontal Meningioma After Localized IMRT on Posterior Meningioma Resection Cavity Without Adjuvant Immunotherapy

**DOI:** 10.3389/fonc.2019.01109

**Published:** 2019-10-18

**Authors:** Danielle Golub, Kevin Kwan, Jonathan P. S. Knisely, Michael Schulder

**Affiliations:** ^1^Department of Neurosurgery, North Shore University Hospital, Zucker School of Medicine at Hofstra/Northwell, Manhasset, NY, United States; ^2^Department of Neurosurgery, New York University School of Medicine, NYU Langone Health, New York, NY, United States; ^3^Department of Radiation Oncology, New York-Presbyterian Hospital/Weill Cornell Medical Center, New York, NY, United States

**Keywords:** abscopal effect, immunotherapy, meningioma, off-target effect, radiation

## Abstract

**Background:** Localized radiation therapy (RT) is known to infrequently cause off-target or “abscopal” effects at distant metastatic lesions. The mechanism through which abscopal effects occur remains unknown, but is thought to be caused by a humoral immune response to tumor-specific antigens generated by RT. Combination treatment regimens involving RT and immunotherapy to boost the humoral immune response have demonstrated synergistic effects in promoting and accelerating abscopal effects in metastatic cancer. Nevertheless, abscopal effects, particularly after RT alone, remain exceedingly rare.

**Case Presentation:** We report the case of an 84-year-old man with an atypical meningioma, who demonstrated a radiographically significant response to an untreated second intracranial lesion, likely also a meningioma, after intensity-modulated radiation therapy (IMRT) to a separate, detatched resection cavity. Serial annual MRI imaging starting at 2- to 3.5-year (most recent) post-IMRT follow-up demonstrated a persistent decrease in both tumor size and surrounding edema in the untreated second lesion, suggestive of a possible abscopal effect.

**Conclusions:** We describe here the first report of a potential abscopal effect in meningioma, summarize the limited literature on the topic of abscopal effects in cancer, and detail the existing hypothesis on how this phenomenon may occur and possibly relate to the development of future treatments for patients with metastatic disease.

## Background

Radiation therapy (RT) has long been established as an effective means of local tumor control. There is increasing evidence supporting additional off-target or “abscopal” effects of RT in metastatic cancers. The abscopal effect was first described and named in 1953 by RH Mole, who demonstrated in animal models that large doses of localized radiation produced organ dysfunction in distant healthy tissues (1). Understanding the underlying biology of both the abscopal effect itself and of tumor immune-escape (in non-responders) is critical to the development of effective therapeutic strategies for metastatic cancers. Preclinical studies suggest that these off-target effects are likely mediated by an anti-tumor immune response primarily involving antigen-presenting cells (APCs) and T-lymphocytes (2).

Clinical studies reporting abscopal effects in humans remain limited. The development of immunomodulatory agents such as cytotoxic T-lymphocyte-associated protein 4 (CTLA-4) and programmed cell death-1/programmed death-ligand 1 (PD-1/PD-L1) inhibitors, however, has recently allowed clinicians to boost the T-cell response to local RT to capitalize on the abscopal effect phenomena and actively treat distant lesions (3). Golden et al. first demonstrated this effect in 2015 in a proof-of-principal trial combining granulocyte-macrophage colony-stimulating factor (GM-CSF) with RT for solid metastatic cancers, and his findings have served as the basis for hundreds of ongoing clinical trials; 27% of patients on Golden's combined regimen demonstrated significant regression at distant metastases and, furthermore, this responding cohort also exhibited prolonged survival compared to those without an abscopal response (4). However, even in this canonical work, the majority of patients treated using this mixed modality approach did not develop a systemic anti-tumor response (4).

Despite advances in immune checkpoint inhibitors as adjuvant therapy and trials of various dosing strategies for fractionated RT in combination, abscopal responses remain rare phenomena. Of particular interest are the exceedingly rare reports of the abscopal effect after RT in which immune modulation therapy was not used (pure “off-target effects” of radiation). Continued study of the molecular, genetic, and phenotypic tumor profiles of these rare responders may contribute to the development of more targeted and personalized combination treatment strategies capable of producing this effect universally.

We herein present the unique case of an 84-year-old man with two dural-based, extra-axial intracranial lesions, one of which demonstrated a significant decrease in size after local RT to the distant surgical cavity of the contralateral lesion, a known atypical meningioma. No adjuvant immunotherapy was administered, making the report of this case a critical contribution to our growing understanding of the abscopal response and treatments for patients with metastatic cancer. Furthermore, this is the first report of a possible abscopal effect in a patient with a meningioma.

## Case Presentation

**Figure 1:**
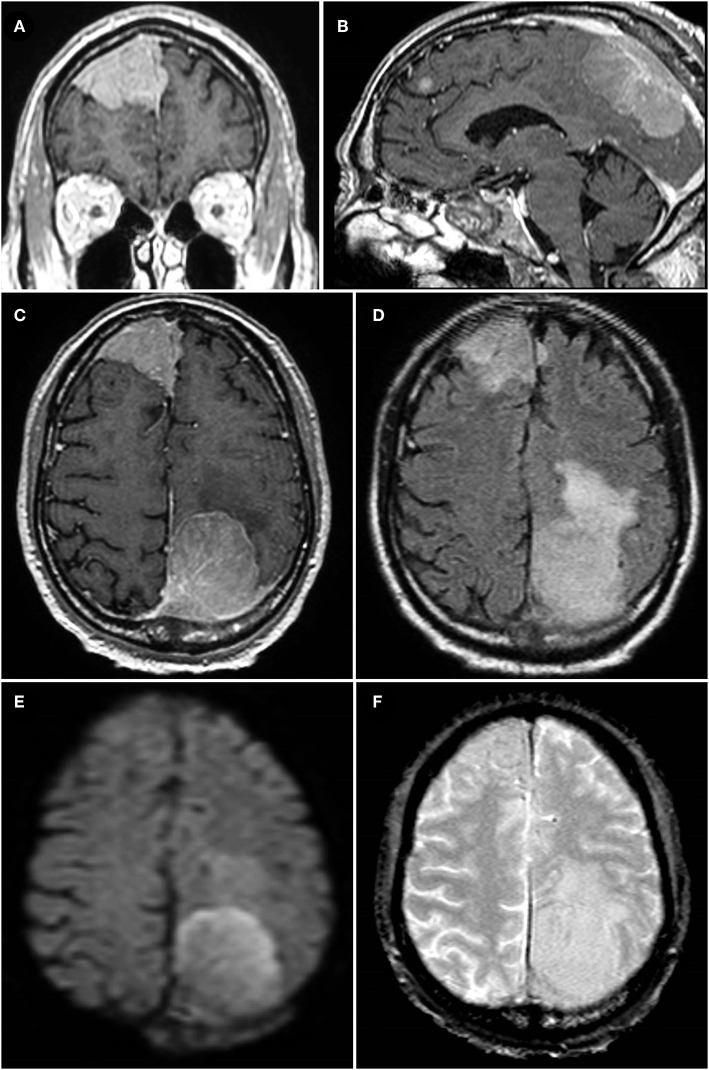
**(A–C)** Coronal, midsagittal, and axial T1-weighted post-contrast MRI images of the patient's two parasagittal, enhancing, extradural lesions; both lesions are seen to invade the superior sagittal sinus, particularly the left parasagittal parietal lesion in **(B)** which also shows evidence of osseous extension. **(D)** Axial T2-FLAIR-weighted sequence showing extensive peri-lesional vasogenic edema of both the right and left parasagittal lesions, worse on the left. **(E)** Diffusion-weighted imaging demonstrating evidence of diffusion restriction within the larger, left parasagittal parietal lesion reflecting high cellular density in the tumor (later determined to be a WHO grade II atypical meningioma). **(F)** Gradient echo (GRE) sequence showing sparsely scattered areas of signal dropout indicative of calcifications, particularly in the right frontal lesion, but no evidence of hemosiderin deposits or other evidence of intratumoral hemorrhage in either lesion.

An 84-year-old male presented to the emergency room after progressively worsening gait instability resulted in a fall. Head CT revealed dural-based parasagittal left parietal and right frontal lesions with scattered calcifications. MRI showed the extra-axial lesions to be contrast-enhancing and most consistent with meningioma, especially given an otherwise negative metastatic workup (Figures 1A–C). Both lesions showed diffusion restriction, surrounding vasogenic edema, and irregular borders (Figures 1D,E). Gradient echo sequence showed no evidence of intratumoral hemorrhage in either lesion (Figure 1F). The left-sided lesion invaded the superior sagittal sinus and adjacent calvarium suggesting intraosseous extension (Figure 1B). The left-sided lesion measured 4.2 × 3.6 × 3.1 cm (transverse, anterior-posterior, and cranio-caudal dimensions) and the right-sided lesion measured 4.5 × 4.3 × 4.2 cm.

The patient initially responded to a short trial of corticosteroids. One year later, he represented with acutely worsened altered mental status, right-sided hemiparesis and sensory loss. MRI showed significantly increased vasogenic edema surrounding the left parietal lesion (now measuring 4.1 × 5.3 × 6.7 cm) with resultant mass effect, midline shift (increased to 1.2 cm from 0.6 cm previously), effacement of the left lateral and third ventricles causing new obstructive hydrocephalus, and effacement of the ambient cistern denoting uncal herniation.

The patient was admitted for urgent craniotomy aimed at subtotal tumor resection given the known invasion of the lesion into the superior sagittal sinus. The portion of the lesion attached to the superior sagittal sinus was left behind. A near-complete resection of the left parasagittal lesion was achieved. Surgical pathology was consistent with an “atypical,” WHO Grade II meningioma.

**Figure 2:**
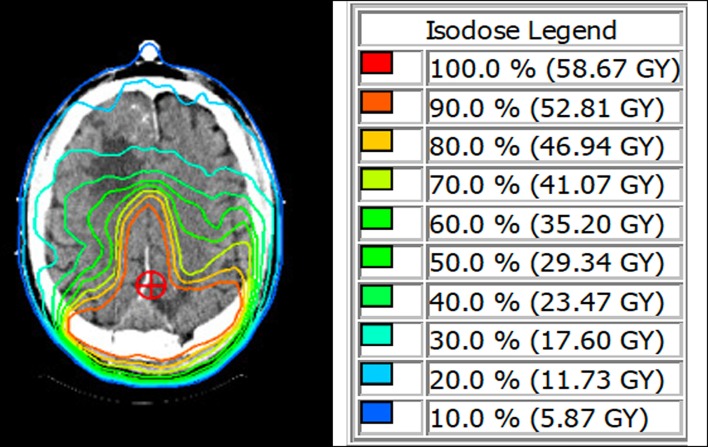
**(Left)** IMRT treatment planning image. Isodose lines are displayed on post-operative axial CT scan. The targeted total dose (red) is shaped around the remainder of the posterior parasagittal meningioma. The right frontal meningioma can be seen within the lighter blue Isodose line, indicating a potential overall dose between 11.7 and 17.6 Gy. **(Right)** Isodose legend with corresponding percent total planned radiation dose and actual radiation dose to be given over 30 fractions.

**Figure 3:**
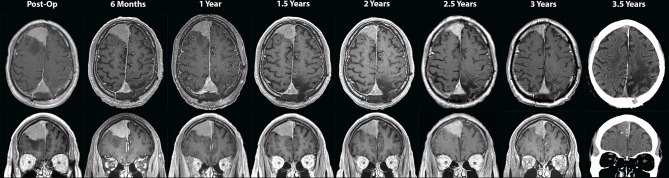
Serial axial **(top panels)** and coronal **(bottom panels)** T1-weighted post-contrast MRI images taken every 6 months during outpatient follow-up, including the initial post-operative scan showing parasagittal parietal meningioma invading the superior sagittal sinus left as residual tumor. Follow-up scans at 6 months, 1 year, and 1.5 years show that the two lesions remained stable in size. On 2-year through the most recently taken 3.5-year scans, regression of both the left parasagittal parietal and the (untreated) right frontal lesions is visualized and attributed to the abscopal effect. A CT scan was performed for follow-up at 3.5 years due to recent placement of a non-MRI-compatible cardiac pacer.

Six weeks after surgery, the patient was started on a fractionated course of intensity-modulated radiation therapy (IMRT) to the left parasagittal post-operative bed. He received a total targeted dose of 54 Gy in 30 fractions over 6 weeks (Figure 2). The right frontal lesion received a subtherapeutic dose between 11.7 and 17.6 Gy over the 30 fractions. He was thereafter followed with serial imaging. MRIs at 6 months, 1 year, and 1.5 years post-treatment were grossly stable (Figure 3). At 2-year follow-up, the left parietal lesion had mildly decreased in size (2.9 × 2.4 × 1.4 cm) and at 2.5-year follow-up, both the left parietal (2.6 × 2.3 × 1.4 cm) and the untreated right frontal (3.5 × 3.1 × 2.6 cm) lesions were measurably smaller. This regression trend continued through to 3 years (L 2.6 × 2.0 × 1.9 cm, R 3.2 × 3.1 × 2.6 cm) and to most recent follow-up with CT scan at 3.5 years (L 2.4 × 2.3 × 1.4 cm, R 3.1 × 3.0 × 1.9 cm), at which point significantly reduced surrounding edema around the right frontal lesion was also noted (Figure 3). Additionally, throughout follow-up, there was no evidence on susceptibility-weighted imaging of hemosiderin deposition indicative of intratumoral hemorrhage at any time. The patient likewise clinically improved; he progressively regained ambulatory stability (now ambulating independently with a cane) and cognition over the course of his follow-up and has been without new neurological complaints.

## Discussion

The abscopal mechanism involves both cellular and humoral immune responses and is most commonly described as follows: Directed radiation triggers local tumor cell death through multiple mechanisms including an immunogenic pathway that involves release of “warning signals” such as damage-associated molecular patterns (DAMPs) and high-mobility group protein B1 (HMGB1) (5). Dendritic cells (DCs), an APC subtype, express toll-like receptors (TLRs) that interpret these danger signals and prompt DC surface expression of tumor-specific antigens. Activated DCs are then responsible for cross-priming cytotoxic T-cell populations for targeted tumor attack. Accordingly, increased activity of T-cell priming significantly increases in draining lymph node tissues adjacent to metastatic disease after RT to primary tumors (6). Likewise, local administration of interleukin-2, a T-cell activating cytokine, has been shown to promote abscopal effects in a mouse model of metastatic rectal cancer (7, 8).

According to RANO criteria (frequently adapted for meningioma) (9), by perpendicular 2-dimensional measurements in the transverse and anterior-posterior directions, the patient's *untreated* right frontal meningioma demonstrated a 30% reduction in size between the baseline MRI and the last follow-up MRI at 3 years, indicative of at least “stable disease” and nearing the threshold for a “partial response” (placement of a non-MRI compatible cardiac device unfortunately prevented further imaging in a consistently measurable manner; subsequent follow-up imaging was performed by CT scan) (10). Observation of an abscopal effect after RT alone (as is hypothesized in this case) is an exceedingly rare phenomenon described only in a handful of reported cases (6, 11–13). Variation in response is likely multifactorial and may involve intra-tumoral heterogeneity, tumor mutational burden, tumor size and localization, patient genetic profile, and differences in radiation dosing strategies. These factors collectively contribute to the nature of the tumor microenvironment, and in a non-responder, the context of a highly immunosuppressive tumor microenvironment likely renders the patient unable to mount an anti-tumor immune response even after tumor antigen exposure (14). Several cancer types (including both solid and hematologic malignancies) have consistently demonstrated a propensity to produce large populations of “suppressor cells,”—in particular, myeloid-derived suppressor cells (MDSCs). MDSCs establish tumor-tolerance locally and systemically by inducing differentiation of FoxP3+ T-regulatory cells (Tregs) that impair cytotoxic T-cell activation by both amino-acid depletion and inhibition of APC/T-cell cross-priming. Tregs also increase tumor and self-expression of PD-L1—directly inducing T-cell apoptosis (15). In preclinical studies, the post-RT abscopal effect has been shown to additionally have some genetic basis; for example, one study in a mouse model of metastatic lung cancer showed the abscopal effect to be dependent on germline p53 status, independent of immune modulation (16). Given that both the abscopal response and tumor immune-escape seem independent of cancer type, studying baseline patient immunity and individual tumor characteristics in abscopal responders, especially in patients who received no immunotherapy, may explain the genetic and tumor-specific predictors of abscopal effects (17, 18).

The premise that the abscopal effect is dependent on a humoral T-cell-mediated response is further supported by the effect's widely reported delay in onset. A robust and recent systematic review of case reports on the abscopal effect described a median time to abscopal effect of 5 months, with a range from starting during RT to over 2 years post-treatment (as seen in our patient) (17). The distribution of time to abscopal effect is exceedingly similar to that of treatment with ipilimumab, a CTLA-4 antibody that enhances cytotoxic T-cell activity in the tumor microenvironment (19). Because of individual variance and months to years-long reported response times, it has been heavily recommended that physicians take caution to not prematurely terminate ipilimumab treatment (20, 21). A need for close longitudinal follow-up of RT-treated metastatic cancer patients is suggested by these similarities. Furthermore, it is hypothesized that the ongoing development of novel antibodies and other immunomodulators, particularly those that target aspects of cellular immunity in parallel with humoral immunity, will enhance or accelerate the abscopal response when given as combination therapy in delayed-responders (22).

In meningioma in particular, both higher grade lesions (WHO grade II or III) and convexity (vs. skull-base) lesions are known to contain a significantly greater intratumoral T-cell infiltrate (23, 24). The clinical and therapeutic implications of this recent finding are 2-fold: (1) Higher grade meningiomas are likely associated with greater local and systemic immune-suppression and tumor immune-escape, particularly grade III lesions which are distinctly associated with increased intratumoral and peripheral PD-L1 expression (23, 25). (2) Both grade II and grade III lesions may therefore be amenable to adjuvant immunotherapy; furthermore, grade II or “atypical” convexity lesions, such as our patient's lesion, which have significant overall T-cell burden but associated low Treg and PD-L1 burden, may more readily demonstrate an immunogenic response solely to a high tumor-specific antigen exposure after RT—an abscopal effect (23). It is well-described that tumors with high somatic mutational burden, or “hot” tumors, tend to generate higher systemic expression of surface neoantigens and thereby provoke a stronger anti-tumor immune response (26). Multiple series have demonstrated that there is a critical subset of grade II and III meningiomas, while still poorly defined, that fall into this category of “hot” tumors exhibiting substantial genetic instability and therefore are more likely to demonstrate positive responses to immunotherapy or even immunogenic responses to RT alone (27, 28). While initial discussion of these potential cases is critical and hypothesis-generating, further molecular characterization of these “responder” tumors is warranted.

Radiotherapy dosing strategies seek to balance the goal of tumor control with an allowance for self-repair of nearby healthy tissues. Our patient received fractionated RT to his post-operative cavity (54 Gy in 30 fractions). Previous work in animal models corroborates that a hypofractionated regimen may be the optimal dosing strategy for achieving an abscopal response. In a mouse colon carcinoma model receiving adjuvant immunotherapy, two fractionated dosing strategies (8 Gy x 3 and 6 Gy x 5) yielded a dramatically superior response compared to a large single dose of 20 Gy. Furthermore, the hypofractionated 8 Gy x 3 regimen was statistically superior to the hyperfractionated regimen both in achieving distant tumor shrinkage and in promoting the development of tumor-specific T-cells (29). While hyperfractionated RT is associated with lower toxicity and may be preferred in high-risk patients with multiple comorbidities, these studies combined with anecdotal case evidence suggest that a hypofractionated regimen is favored in metastatic disease (30). The benefit of a fractionated regimen is thought to be related to the consequent development of a distinct genetic and molecular signature in tumor cells. In particular, interferon-related genes and TGF-beta-related genes are differentially expressed in tumor cells after a fractionated treatment regimen compared to single-dose (31). These findings further support the premise that tumor heterogeneity and mutational burden may play a significant role in differentiating responders and non-responders. The role of these genes in modulating the tumor microenvironment or a systemic immune response after RT remains poorly defined.

Given the patient's history of RT and the timeline being appropriate, it is possible that the tumor regression observed in this case is secondary to an abscopal effect. However, there have been a handful of cases of completely spontaneous regression (regression after no RT, surgical or directed medical intervention) reported in patients with meningiomas under specific circumstances (32–39). Progesterone receptors are expressed in around 70% of meningiomas and their activation is hypothesized to be required for maintenance of meningioma vascular density (38, 40). Spontaneous meningioma regression either after cessation of hormone therapy, particularly progesterone or related analogs, or during the post-partum period are the most well-described regression phenomena (32–35, 38). Additionally, meningioma growth (and consequently regression after cessation of therapy), particularly in men, is known to be associated with androgen deprivation therapy for prostate cancer (41, 42). Intratumoral hemorrhage preceding spontaneous regression is also well-documented both in meningioma and across other tumor types—benign or malignant (36, 43, 44). In the only two reported cases in which hormonal therapy, pregnancy or hemorrhage did not play a role, the patients were female and of perimenopausal/post-menopausal age and decreased circulating hormone levels likely contributed to tumor regression (37, 39). Additionally, these two patients both had uncontrolled diabetes and the authors suggested that associated microangiopathic changes may have played a role in tumor devascularization and shrinkage (37, 39). Lastly, while there may be some contribution of resolving edema on the tumor volume itself, this effect would most likely be minimal as significant tumor contraction attributable to decreased edema has only been reported in high grade meningioma with adjunctive systemic anti-angiogenic treatment (bevacizumab) (45). In summary, our patient—an elderly male—was never on hormonal therapy for any previous medical condition (i.e., no history of prostate cancer) or during his tumor treatment course, did not have microvascular disease risk factors, and serial imaging (performed routinely with susceptibility-weighted sequences) never showed evidence of intratumoral hemorrhage preoperatively or during longitudinal follow-up making a post-RT abscopal effect a likely etiology for regression of his second lesion.

Another consideration, however, is the lack of histologic confirmation of meningioma for the second regressed frontal lesion. The differential diagnosis of an extra-axial, dural-based, supratentorial, enhancing lesion includes meningioma (most common), metastasis, as well as some potentially more radiosensitive lesions—leptomeningeal hemangioblastoma and hemangiopericytoma (46). Metastasis was unlikely given negative metastatic workup, clinical features of hemangioblastoma such as a history of von Hippel-Lindau (VHL) and polycythemia were not present, and imaging features associated with hemangiopericytoma, such as lytic bone lesions and lack of calcifications, were not consistent with our patient's MRI findings (47–50). Furthermore, supratentorial hemangioblastoma without VHL is exceedingly rare, with only about 60 total reported cases, and hemangiopericytoma likewise only represents 0.4% of primary intracranial tumors, making meningioma the most likely pathology based on clinical and imaging features (46, 51). Additionally, multiple intracranial meningiomas are known to occur in up to 10% of patients with diagnoses of meningioma, a much higher incidence than both primary hemangioblastoma and hemangiopericytoma (52). With regards to the radiated field, while the right frontal lesion did receive up to 11.7 Gy (~20% of the total radiation dose), it is highly unlikely that this subtherapeutic dose directly influenced tumor regression given that the lowest reported efficacious fractionated IMRT dose for tumor control in atypical meningioma is 54 Gy (53, 54).

## Conclusion

This case is critically hypothesis-generating as it presents the first report of a possible radiation-only induced abscopal effect in meningioma. The off-target, systemic immune response was likely kindled by fractionated IMRT alone, without adjuvant immunotherapy. Further study of the genetic and molecular characteristics of patients who develop abscopal responses either without adjuvant immunotherapy (as in this case) or within a rapid time course is warranted for development of more targeted neuro-oncological treatment modalities, particularly in patients with higher grade meningiomas.

## Data Availability Statement

The datasets generated for this study are available on request to the corresponding author.

## Ethics Statement

Written informed consent regarding the submission and potential publication of this manuscript was obtained from the case study patient. Additionally, consent for treatment was likewise obtained in the usual fashion during the course of the patient's hospitalization. As a single case report study, institutional review board review was not required for this study by the authors' home institutions. Written informed consent for participation was not required for this study in accordance with the national legislation and the institutional requirements.

## Author Contributions

DG and KK: manuscript drafting and literature review. JK and MS: clinical data collection, interpretation, research team management, and oversight. DG, KK, and MS: figure design. DG, KK, JK, and MS: manuscript editing and revision.

### Conflict of Interest

The authors declare that the research was conducted in the absence of any commercial or financial relationships that could be construed as a potential conflict of interest.
